# Parental depression moderates the relationship between childhood maltreatment and the recognition of children expressions of emotions

**DOI:** 10.3389/fpsyt.2024.1374872

**Published:** 2024-06-05

**Authors:** Annie Bérubé, Rachel Pétrin, Caroline Blais

**Affiliations:** ^1^ Ricochet, Department of Psychoeducation and Psychology, University of Quebec in Outaouais, Gatineau, QC, Canada; ^2^ Centre de Recherche Universitaire sur les Jeunes et les Familles (CRUJeF), Trois-Rivières, QC, Canada; ^3^ Social and Visual Perception Laboratory, Department of Psychoeducation and Psychology, University of Quebec in Outaouais, Gatineau, QC, Canada

**Keywords:** childhood maltreatment, emotion recognition, depression, parental sensitivity, parent-child relationship

## Abstract

**Background:**

Sensitivity plays a crucial role in parenting as it involves the ability to perceive and respond appropriately to children’s signals. Childhood maltreatment and depression can negatively impact adults’ ability to recognize emotions, but it is unclear which of these factors has a greater impact or how they interact. This knowledge is central to developing efficient, targeted interventions. This paper examines the interaction between parents’ depressive symptoms and childhood maltreatment and its influence on their ability to recognize the five basic emotions (happiness, anger, sadness, fear, and disgust) in children’s faces.

**Method:**

The sample consisted of 52 parents. Depressive symptoms were measured by the depression subscale of the Brief Symptom Inventory-18 (BSI-18), and maltreatment history was assessed by the Childhood Trauma Questionnaire (CTQ). Children’s emotional stimuli were morphed images created using The Child Affective Facial Expression (CAFE) database.

**Results:**

Our findings indicate that depressive symptoms moderate the relationship between parents’ history of childhood maltreatment and emotion recognition skills. Parents with higher depressive symptoms had lower emotion recognition accuracy when they had not experienced maltreatment. When childhood maltreatment was severe, emotion recognition skills were more consistent across all levels of depression. The relationship between depression and emotion recognition was primarily linked to recognizing sadness in children’s faces.

**Conclusion:**

These findings highlight how different experiences can affect parental abilities in emotion recognition and emphasize the need for interventions tailored to individual profiles to improve their effectiveness.

## 1 Introduction

Childhood maltreatment can have long-lasting consequences on a person’s development. It can also affect their ability to become a parent. Studies have found that individuals who experienced maltreatment during their childhood may face more challenges in being sensitive parents (1). The experience of maltreatment during childhood is also related to mental health and has been repeatedly related to depression (for a review, see 2). While parental experience of maltreatment and depression are known to be linked to sensitivity, further research is needed to understand the underlying mechanisms. Our study contributes to this understanding by examining how childhood maltreatment and depressive symptoms are related to an essential aspect of sensitivity: recognizing children’s emotional signals.

Individuals who have experienced maltreatment during childhood are at high risk of developing mental health difficulties. Several systematic reviews and meta-analyses have confirmed the link between a history of maltreatment and depression (2, 3). According to a meta-analysis, experiencing childhood abuse and neglect raises the odds ratio of developing depression by 1.5 to 2.5, depending on the type of maltreatment experienced (4). This association is seen in both men and women, with women showing a larger effect, although not significantly so (5). Many mediators have been used to explain the development of depressive symptoms following childhood maltreatment experiences. Examples include envy (6), loneliness and lack of coping skills (7), scarcer social support (8), and lower parent-child relationship quality (9). Recent research has focused more on emotion dysregulation, which refers to an individual’s inability to control or regulate their emotional responses to a stimulus (10–13).

Effective emotional regulation involves four key dimensions: understanding and being aware of emotions, accepting emotions, being able to engage in goal-oriented behavior and avoid impulsive behavior, and using appropriate strategies to regulate emotions (14). Depression has been found to play a significant role in shaping differences in emotion recognition (15, 16). Abnormal processing of facial expressions of emotions has been linked with mental health disorders. However, research suggests these deficits are more common in individuals with Major Depressive Disorder (MDD) than those with anxiety disorders (17). One possible explanation for this impairment might be the presence of negative thoughts and beliefs in MDD, such as worthlessness and self-criticism. These negative cognitions could lead to a more negative evaluation of external stimuli, including facial expressions, compared to healthy individuals (18). A meta-analysis indicates that individuals suffering from MDD have more difficulty recognizing the emotions of anger, disgust, fear, happiness, and surprise (19). Moreover, the ability to recognize emotional facial expressions is affected by the severity, not just the presence, of depressive symptoms. Krause et al. (20) found that participants with MDD who had severe depressive symptoms and were admitted to inpatient facilities showed less accuracy in identifying happy emotional facial expressions than those with mild symptoms attending outpatient facilities.

Individuals who have undergone maltreatment in the past also face challenges in managing their own emotions, as well as having difficulties accurately perceiving and interpreting the emotions expressed by others (21). The association between childhood maltreatment and emotion recognition is well-established, and its long-lasting consequences have been documented at various stages during a person’s lifetime. Pollak et al. (22) were among the first to demonstrate how different forms of maltreatment affected emotion recognition in children (see (23), for a systematic review). Another systematic review found that emotion recognition continued to be altered during adulthood (24).

The role of depression in the relationship between childhood maltreatment and emotion recognition has been explored. According to Hautle et al. (25), individuals with a history of maltreatment and a mental disorder diagnosis have the lowest levels of emotion recognition accuracy, followed closely by those with childhood maltreatment but without a diagnosis. On the other hand, participants with a diagnosis but no history of maltreatment have the highest levels of emotion recognition accuracy. In a study by Suzuki et al. (26), adults with a history of maltreatment made more errors in recognizing fear than anger compared to depressed adults with a history of maltreatment who showed an increased bias towards fear.

Three comparable studies have shown that adults diagnosed with MDD who have experienced childhood maltreatment tend to have more difficulty recognizing emotions than MDD-diagnosed adults who did not experience childhood maltreatment. All of these used the Reading the Mind in the Eyes (RMET) task to evaluate participants’ emotion recognition abilities, which involves matching mental state descriptors with an image of the eye region of an adult’s face (27). Rnic et al. (28) found that individuals with a history of emotional abuse who were suffering from MDD had a significantly lower accuracy rate in the RMET. However, those with a history of physical abuse in the non-depressed group had no significant difference in their RMET scores. They also noticed that a history of neglect enhanced the accuracy rate in depressed and non-depressed groups. Similarly, (29) documented that childhood emotional and physical neglect negatively affected participants’ response accuracy to the RMET task compared to controls without MDD. When combined with early-life neglect, emotional abuse specifically interfered with the accuracy of the positive and negative valences. The number of childhood adversities predicted total and negative valence RMET scores. A more recent study using the RMET by Nilsson et al. (30) revealed that patients with MDD and a history of childhood maltreatment had poorer emotion recognition abilities than MDD patients without such a past. The difference was observed in decoding positive and negative emotions, while no significant group differences emerged for decoding neutral emotions. Emotional neglect was the only maltreatment type that was found to be associated with lower emotion decoding abilities. In summary, mixed results were obtained regarding the impact of maltreatment and depression on emotion recognition and the valence of emotions that each form of maltreatment can influence.

Previous studies have mainly focused on exploring the role of depression in the relationship between childhood maltreatment and emotional recognition in MDD-diagnosed adults, using adult facial stimuli to measure emotion recognition accuracy. Little attention has been given to understanding how the presence and severity of depressive symptoms can affect parents with a history of maltreatment in recognizing children’s emotional facial expressions.

Accurately recognizing children’s facial expressions of emotions is a crucial parenting component. During infancy, parents rely on their baby’s cries to know and meet their needs. As children grow older, they develop more complex ways to express their needs. Preschoolers, for instance, cry less often but do not have the whole vocabulary to explain their needs. During this phase, parents need to identify the emotional signals that their children communicate through facial expressions and adapt their responses accordingly. Therefore, this paper investigates how depression interacts with parents’ history of childhood maltreatment to influence their ability to recognize the six basic emotions (happiness, anger, sadness, fear, disgust, and surprise) in children’s faces.

## 2 Materials and methods

The study received approval from the Ethical Committee of the University of Quebec in Outaouais (UQO CER #2020-698) and was conducted between January 2020 and October 2023 by the university’s guidelines and regulations. All participants provided written informed consent before their involvement in the study.

### 2.1 Participants

The study involved one parent from 52 families: 46 identified as women and 6 as men (*M age* = 35.5 years, *SD* = 6.0). Each parent was accompanied by one of their preschool children aged between 2 and 5 years (*M* = 53.2 months, *SD* = 15.2, 50% female). A power analysis revealed that a sample of 52 participants with two predictors and three control variables allowed an effect size of.20 with a power of.80. Participants were recruited from local community organizations that provide services to vulnerable families, university bulletin boards, and social media. Most were Caucasian (82.7%) and had completed a university degree (53.8%). However, 7.7% of parents had completed primary school, and 23.0% had completed secondary school or a vocational diploma. About 53.8% of the parents had an annual income of 70 000 CAD or more compared to 26.9% with an income of 23 999 CAD or less. A total of 16 participants (30.8%) indicated having experienced at least one form of maltreatment at a severity level from moderate to extreme. Scores on the depression scale indicated that 44.2% of parents had a low level of depression (raw score of 0, t-score 41), and 19.2% had a high level of depression (raw score of 5 and above, t-score ≥ 63) (see **Table 1**).

**Table 1:** Sociodemographic characteristics of parents (n = 52).

Variables	Frequency	Percentage (%)
Parent’s gender Female Male	46 6	88.5 11.5
Parent’s education level Primary Secondary and Vocational studies College University	4 12 8 28	7.7 23.0 15.4 53.8
Parent’s income (in CAD) 0 to 23 999$ 24 000 to 39 999$ 40 000 to 69 999$ 70 000 and above	14 5 5 28	26.9 9.6 9.6 53.8
Parent’s ethnicity Caucasian Non-Caucasian	43 9	82.7 17.3
Parent’s CTQ scores None to little maltreatment Moderate to extreme maltreatment	36 16	69.2 30.8
Parent’s depressive symptoms None (t-score 41) Moderate (t-score 50 to 61) Clinical level (t-score 63 and above)	23 19 10	44.2 36.6 19.2

### 2.2 Procedure

This study is part of a more extensive protocol. Only the tasks related to the current study are described here. Participants were asked to consent to a two-hour study before the experiment. Children and their parents were taken to separate rooms. Parents did various computer tasks, among which one asked them to identify the most prominent emotion on images morphing two facial expressions of children showing emotions at different intensities. After that, the parents and their children were reunited for an observational task. They were asked to interact for 15 minutes. The first 7 minutes was a free play period, after which the parents had to ask their children to clean the room without support other than verbal encouragement. After the interaction, the parents and children were separated again, and the parents were asked to complete questionnaires. Parents received a reward of 40$ CAN for their participation, and the children received a gift such as a book or puzzle.

### 2.3 Measures

#### 2.3.1 Demographic information

Parents provided demographic information through a self-reported questionnaire that includes questions regarding the child’s gender and age, the parents’ age, ethnicity, education, family status, annual income, and other pertinent information (31).

#### 2.3.2 Childhood maltreatment

The French version of the Childhood Trauma Questionnaire (CTQ) was used to determine if the parents had experienced any maltreatment in their childhood (32). The CTQ is a self-reported questionnaire consisting of 28 questions that use a Likert-type scale ranging from 1 (*Never true*) to 5 (*Very often true*). The CTQ evaluates childhood maltreatment in five forms: emotional neglect, physical neglect, emotional abuse, physical abuse, and sexual abuse. A score is given for each type of maltreatment experienced by the individual. The CTQ has been validated with a large population and has good test-retest reliability (r between 0.76 and 0.96). The short version of the CTQ (28 items) has been validated by multiple studies (32, 33) and can be used with a French-speaking population. For each type of maltreatment, a score is calculated to determine the severity of the reported experiences, where 1 indicates no maltreatment, 2 indicates low maltreatment, 3 indicates moderate maltreatment, and 4 indicates severe maltreatment. A total severity score was calculated for our study by adding the severity score for each form of maltreatment. The total severity score can range from 5 to 20. The internal consistency for our sample is α = .85.

#### 2.3.3 Depressive symptoms

The depression subscale of the Brief Symptom Inventory-18 (BSI-18) was used in this study (34). The BSI-18 is a shortened version of the BSI developed by Derogatis and Melisaratos (35). The depression subscale consists of 6 items rated on a Likert-type scale ranging from 1 (*Not at all*) to 5 (*Extremely*). Participants were asked to rate the extent to which they had been “distressed or bothered” by each symptom during the previous week, and their responses were scored from 0 to 4. The scores of each item were then added up to calculate the total score for depressive symptoms. Scores were transformed in T-scores to facilitate interpretation. The BSI has been validated in many populations (36, 37) and has excellent test-retest reliability (ranging from.68 to.91) and good internal consistency reliability (coefficients ranging from.71 to.85) (35). Derogatis (38) administered both the BSI and SCL-90-R to a sample of 565 outpatients and found that similar symptom dimensions on the two tests correlated highly, with correlations ranging from.92 to.98. The internal consistency of the BSI-18 has been demonstrated by two studies (34, 39). The internal consistency for our sample is α = .85.

#### 2.3.4 Emotion recognition

The stimuli used were photographs of two Caucasian children, a 4.6-year-old boy and a 5.3-year-old girl, expressing six emotions (anger, disgust, fear, joy, sadness, and surprise). These images were obtained from the Child Affective Facial Expression (CAFE) database (40) and were about 13 cm by 13 cm in size. They were presented on a computer screen in a random order. The images were grayscale with a neutral background, and the faces were approximately 13 degrees of visual angle. The emotional expressions were combined in pairs using morphing software (Fantamorph 5.0) with specific weightings (20:80, 35:65, 50:50, 65:35, and 80:20%), resulting in 150 stimuli (15 expression combinations x 2 genders x 5 weights). Parents participated in 450 trials, which were divided into three equal blocks to minimize fatigue. The task was to identify the dominant emotion in each displayed stimulus. Correct responses were defined as identifying the emotion with a weight of 50% or more. No feedback was provided on the accuracy of the answer. This task has been previously validated and used in other studies (41).

Based on unbiased hit rates, the scoring method followed Wagner’s (42) approach. Unbiased hit rates considered both hits and false alarms, with the proportion of hits multiplied by the ratio of hits to the total number of times a participant categorized any stimulus as corresponding to the target emotion. To account for proportions unbiased hit rates underwent arcsine transformation. The average unbiased hit rates across the six emotions represented each participant’s score. The internal consistency of the scoring system, measured by Cronbach alpha, was 0.69, indicating coherence while highlighting variations in performance across different emotions.

### 2.4 Statistical analyses

Descriptive and correlational analyses were performed to test the linear relationship between maltreatment, depression, and emotion recognition. Demographic variables were examined to determine their potential confounding effect. The moderation role of depression was verified by using a multiple linear regression model. The model comprises maltreatment, and depression, as well as their interaction term: maltreatment X depression.

The analyses were performed using SPSS 29.0.0 with the command PROCESS 3.1, model 1 (43). To control for homoscedasticity, the macro provides a heteroskedasticity-consistent standard error (HCSE) estimator of ordinary least squares (44). We used the HC0 (Huber-White) as the HCSE estimator (45). Education level, and gender were included in the models as covariates, as they are correlated to either depression (education), or emotion recognition (gender, and education). To explain the nature of the interactions, simple slope analyses were performed with slopes conditioned at low (-1 SD), moderate (mean), and high (+1 SD) levels (46).

## 3 Results

**Table 2** presents the descriptive statistics and bivariate correlations for all variables. The correlation analysis showed a difference in how mothers and fathers perceived children’s emotional facial expressions (r = -.39; p <.001), with mothers having better recognition scores than fathers. Therefore, gender was considered a control variable. We also conducted a moderation analysis, which excluded fathers, to confirm that their presence did not explain our results. The same results were found with and without including fathers (see **Supplementary Material**). As a result, we decided to include fathers in the results presented below. Parents’ level of education was positively correlated with emotion recognition total scores (r = .41; p <.01) and negatively correlated with depression scores (r = -.31; p = .03). In other words, the more educated the parents, the better they were at recognizing children’s emotional facial expressions, and the fewer depressive symptoms they experienced. Parent’s annual income was positively associated with total emotion recognition scores (r = .30; p = .03). The higher their income, the better they were at recognizing children’s emotional facial expressions. Finally, parents’ recognition scores for surprise were positively related to total CTQ group scores (r = .32; p = .02). Thus, parents who recognized children’s facial expressions of surprise more accurately had higher scores of childhood maltreatment.

**Table 2:** Bivariate correlations and descriptive statistics for each study variable (n = 52).

Variables	1	2	3	4	5	6	7	8	9	10	11	12	13	14	15	Min	Max	Mean	SD	Skewness	Kurtosis
**1. Parents’ age (years)**	1.00															22.0	53.0	35.5	6.01	.26	.66
**2. Children’s age (months)**	.47**	1.00														30.0	84.0	53.2	15.2	.14	-.96
**3. Parents’ gender**	.27	.12	1.00													1.00	2.00	–	–	2.48	4.3
**4. Parents’ education level**	.41**	.13	-.23	1.00												1.00	5.00	–	–	-.86	-.87
**5. Parents’ income**	.27	.11	-.10	.69**	1.00											1.00	7.00	–	–	-.60	-1.56
**6. Parents’ ethnicity**	.23	.23	.15	.07	-.03	1.00										1.00	2.00	–	–	1.78	1.21
**7. CTQ group scores**	.07	.20	-.10	-.11	-.09	.36**	1.00									5.00	18.0	7.44	3.54	1.77	2.35
**8. t-score BSI depression**	-.12	.07	-.09	-.31*	-.01	-.19	.21	1.00								41.0	76.0	51.4	11.1	.62	-.83
**9. Emotions total**	-.09	-.09	-.39**	.41**	.30*	.28*	.02	-.11	1.00							.40	.86	.66	0.11	-.39	-.40
**10. Anger**	-.13	-.13	-.34*	.23	.05	-.34*	-.06	-.07	.76**	1.00						.39	1.06	.81	.15	-.57	-.27
**11. Disgust**	-.05	-.01	-.29*	.28*	.25	-.23	-.03	.03	.73**	.56**	1.00					.05	.78	.34	.19	.42	-.70
**12. Fear**	-.09	-.02	-.28*	.27*	.30*	-.01	-.06	-.27	.67**	.29*	.31*	1.00				.12	.85	.41	.18	.53	-.19
**13. Happiness**	-.05	-.14	-.27	.30*	.17	-.22	.04	-.02	.71**	.48**	.34*	.29*	1.00			.73	1.29	1.12	.14	-.96	.56
**14. Sadness**	-.06	-.07	-.34*	.38**	.23	-.38**	-.02	-.17	.85**	.63**	.55**	.54**	.59**	1.00		.19	.99	.70	.17	-.83	.73
**15. Surprise**	.05	-.05	-.07	.28*	.22	.03	.32*	.09	.47**	.22	.15	.27*	.41**	.20	1.00	.37	.86	.61	.12	-.05	-.50

SD, Standard deviation.

**. Correlation is significant at the 0.01 level (two-tailed). *. Correlation is significant at the 0.05 level (two-tailed).

Multiple hierarchical regression analyses were performed to examine the moderating role of depressive symptoms on the relationship between a history of childhood maltreatment and emotion recognition. The model was significant *F*(5, 46) = 5.65, *p* < 0.01, with variables accounting for 29,97% of the variance in emotion recognition. The interaction term Maltreatment X depression significantly predicted emotion recognition (*b* = .002, t(46) = 2.59, *p* = .01) (**Table 3**).

**Table 3:** Moderated regression analyses predicting parents’ ability to recognize **emotions** in children’s faces (total score; n=52).

	Estimate	SE	95% CI		*p*
			LL	UL	
Constant	.67	.06	.55	.79	.0000
Maltreatment (X1, centered)	.001	.003	-.008	.01	.82
Depression (X2, centered)	-.003	.004	-.01	.005	.46
Interaction X1*X2	.002	.001	.0004	.004	.01
Education Level	.03	.009	.009	.05	.004
Gender	-.11	.03	-.17	-.04	.002

**Figure 1:**
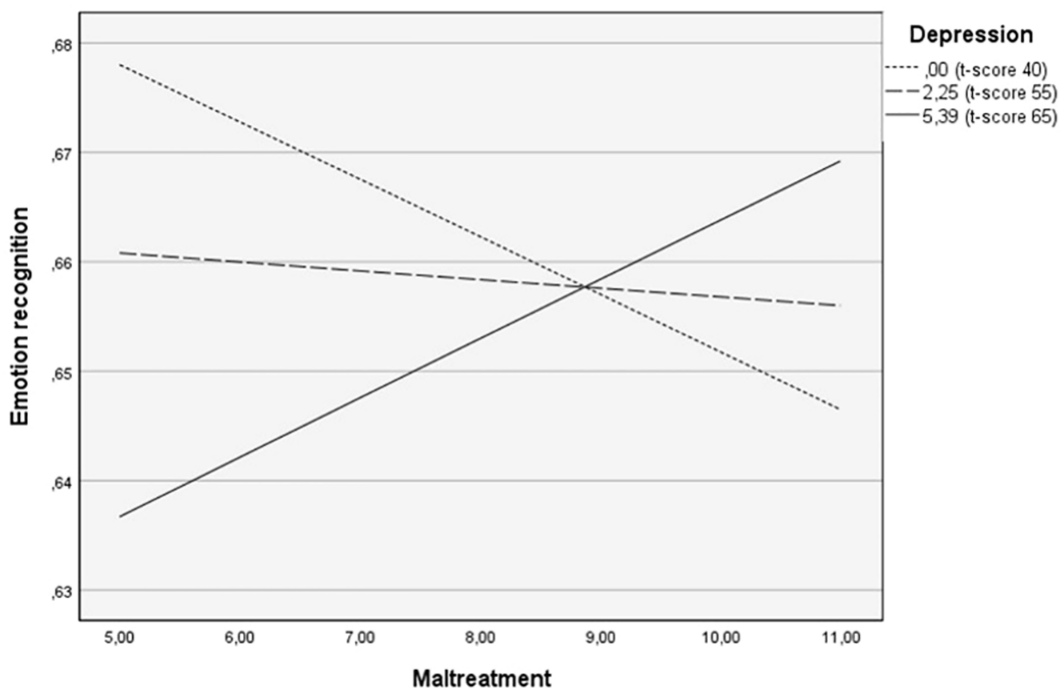
The moderating role of depression in the relationship between childhood maltreatment and overall emotion recognition in children’s faces.

The Johnson-Neyman technique was used to determine the depression score at which the slopes became significant in predicting emotion recognition based on the interaction between depression and a history of maltreatment. Results showed that when the depression score was at a clinical level of 69 or above, the interaction between a history of maltreatment and depression significantly predicted emotion recognition (for a raw score greater than 7.6, t-score ≥ 69: *b* = 0.01, *t*(46) = 2.01, *p* = .05). To represent the interaction, a simple slope test analysis was performed, where depression level was computed at low (-1 SD; corresponding to a t-score of 41), moderate (mean; t-score of 55), and high (+1 SD; corresponding to a t-score of 65) levels. Parents with higher depressive symptoms and no history of maltreatment had lower recognition accuracy. However, their emotion recognition abilities improved with increasing severity of maltreatment. When the severity of maltreatment was severe, their performance became less differentiated, as shown in **Figure 1**.

Exploratory analyses were conducted to identify the emotion that contributed the most to the interaction between maltreatment and depression. Benjamini-Hochberg procedure was applied to control for the false discovery rate. The critical and corrected p-values are presented for each regression model tested. The analysis is deemed significant only when the p-value is below the corrected values, and both are under .05. Among all the regression models tested, the only significant model with a significant interaction effect was the one predicting sadness, whereas, for anger, the model was significant with a marginal interaction (*p* =.058). Only the results for sadness are presented below.

For sadness, the model was found to be significant *F*(5, 46) = 3.42, (*p* = .01, *adjusted-p* =.015), with the variables accounting for 25.54% of the variance. The interaction between maltreatment and depression significantly predicted parents’ ability to recognize sadness (*b* = 0.003, *t*(46) = 2.48, *p* = .02), as shown in **Table 4**.

**Table 4:** Moderated regression analyses predicting parents’ ability to recognize **sadness** in children’s faces (n=52).

	Estimate	SE	95% CI		*p*
			LL	UL	
Constant	.71	.11	.49	.92	.000
Maltreatment (X1, centered)	-.003	.004	-.01	.005	.48
Depression (X2, centered)	-.006	.006	-.02	.006	.29
Interaction X1*X2	.003	.001	.0006	.006	.02
Education Level	.04	.01	.007	.07	.02
Gender	-.15	.06	-.28	-.01	.03

**Figure 2:**
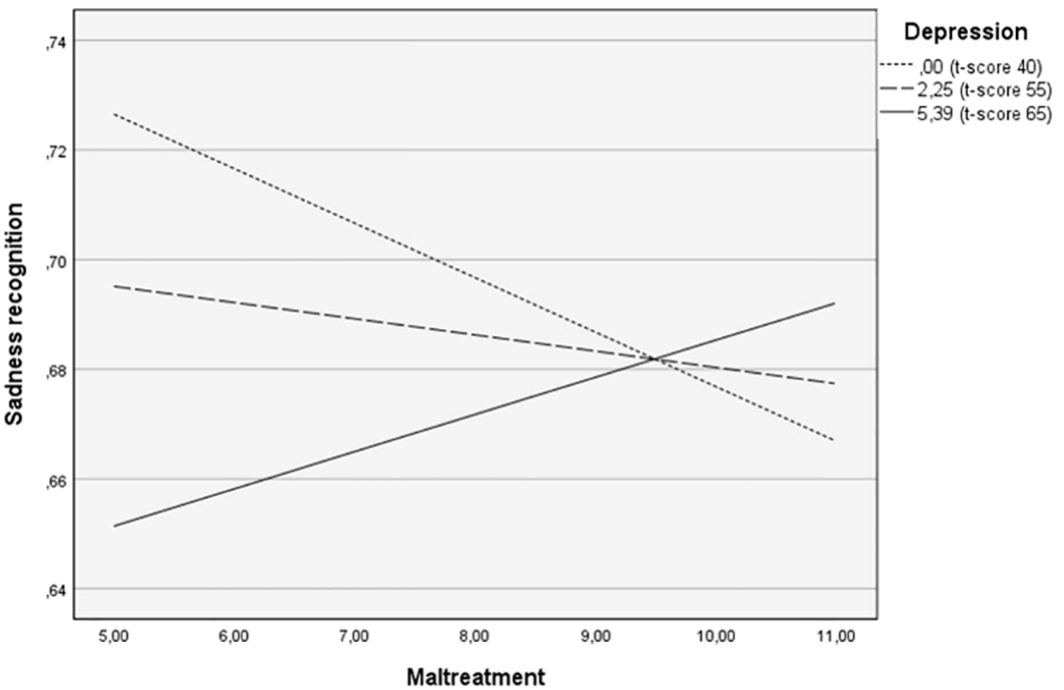
The moderating role of depression in the relationship between childhood maltreatment and sadness recognition in children’s faces.

The Johnson-Neyman technique indicated that at a depression score of 72 and above, the interaction between a history of maltreatment and depression significantly predicted sadness (for a raw score of 9.32 or higher, t-score ≥ 72: *b* = 0.02, *t*(46) = 2.01, *p* = .05). Simple slope test analysis was performed with depression levels at low (-1 SD; corresponding to a t-score of 41), moderate (mean; t-score of 55), and high (+ 1 SD; to score of 65) levels. For parents without depression symptoms, a history of maltreatment is related to a poorer recognition of sadness. However, for parents who combine higher depressive symptoms with a history of childhood maltreatment, the severity of maltreatment is related to an increased ability to recognize sadness. At a severe level of maltreatment, the recognition abilities show less differentiation between all levels of depression (see **Figure 2**).

## 4 Discussion

Both childhood trauma and depression can lead to important socioemotional difficulties, especially in the context of parenting. A history of childhood maltreatment is considered one of the most important predictors of child abuse and neglect in the next generation (47–50). Maternal depression is also an important risk factor for low parental sensitivity (51–53). One possible explanation for this shared prognostic is difficulties with emotion recognition.

The purpose of this study was to investigate the impact of childhood maltreatment and depressive symptoms on parents’ ability to recognize children’s emotional facial expressions. Our findings indicate that when considered together, childhood maltreatment and depressive symptoms have a significant impact on parents’ emotion recognition skills. More specifically, we found that parents with lower levels of depression who experienced severe childhood maltreatment had lower emotion recognition accuracy. On the other hand, parents with higher levels of depression had low emotion recognition abilities when there was no maltreatment. However, their abilities tended to increase as maltreatment severity increased. As the severity of maltreatment increases, the difference between the emotion recognition skills of parents with different depressive profiles decreases. Our results are consistent with Hautle and colleagues’ study (25), which found that participants with a history of maltreatment had lower recognition abilities than those without a traumatic childhood. In their research, mental disorders did not significantly affect recognition abilities for neutral or negative emotions. In concordance with our study, childhood maltreatment led to similar performance outcomes across different mental disorder profiles.

That said, the study of Hautle and al.’s (25) did not examine the combination of no maltreatment history with a mental disorder. Our study provides this information and suggests that individuals with high levels of depressive symptoms but without a history of maltreatment are more likely to have lower emotional recognition abilities. Our study contributes to the existing research by analyzing how depression levels are impacted by the severity of childhood maltreatment, using continuous data instead of grouping individuals into categories. Unlike previous studies that focused on adults diagnosed with major depression and having experienced childhood maltreatment or not, our sample included parents with varying degrees of childhood maltreatment severity and diverse levels of depressive symptoms. Our findings suggest that when there is no history of maltreatment, depression levels are linked to lower accuracy in emotion recognition. However, for individuals with depressive symptoms, the accuracy of emotion recognition increases with the severity of maltreatment.

Another particularity of our study is that instead of using adult faces, we exposed participants to children’s facial expressions of emotions. This could explain the discrepancy between our results and the ones from studies that employed the RMET paradigm, which requires participants to recognize emotions from pictures of adults’ eyes. For instance, Rnic et al. (28) found that depressed participants consistently performed with less accuracy when recognizing negative expressions in the eyes. Contrary to our results, however, they found that participants exposed to depression and emotional abuse performed more poorly on negative valence emotions than the other groups. Similarly, in Nilsson et al.’s (30) study with a group of clinically depressed participants, those with childhood maltreatment had a worse performance with positive and negative emotional expressions in the eyes than participants without maltreatment. Very few studies have explored parents’ ability to recognize emotions through children’s faces. Current findings reveal that parents who have experienced maltreatment in the past exhibit no difference in their reactions to positive, neutral, or negative expressions on infant’s faces. However, this blunted reaction is not observed when they look at adult faces, as reported by Olsavsky et al. (54). Therefore, it may be crucial to test participants with children’s faces to fully understand the role of emotion recognition in the relationship between childhood maltreatment and parenting.

Our results were primarily influenced by the accuracy of identifying sadness in children’s faces. We found that parents who had high levels of depression had greater difficulty in recognizing sadness, especially if they had no history of maltreatment. On the other hand, when childhood maltreatment was severe, the accuracy of recognizing sadness was similar across all levels of depression. Our results are consistent with a review conducted by Bourke et al. (15), which suggests that individuals with major depression typically exhibit lower accuracy in recognizing both sadness and happiness. In their study, ambiguous responses were more likely to be perceived as negative emotions, and neutral expressions were often misinterpreted as sad. Bourke’s results (15) indicate a persistent bias towards sadness in various research paradigms. In a more recent study, participants with major depressive disorder and a history of maltreatment had more difficulty identifying sadness compared to other emotions. Their reaction times were significantly slower than healthy control participants in a matching task that asked them to identify an adult face like the one presented on the top of the screen (55). However, these findings contradict the results of Dalili and colleagues’ (19) meta-analysis, which showed that depressed individuals had difficulty recognizing all emotions, except for sadness. There are two possible explanations for these contradictory results. Firstly, when depression and childhood maltreatment interact, the outcome is different than when either of these variables is considered individually. Secondly, the conflicting results between Dalili et al. (19) and Bourke et al. (15) emphasize the importance of using an unbiased correction when measuring emotion recognition. Participants may be biased toward certain emotions and choose them even when they are not present, which can lead to incorrect results. Unbiased scoring corrects for such misattributions and decreases the accuracy of biased emotions. Bourke et al. (15) accounted for this in their study by evaluating tasks separately if they could lead to misclassification of emotions. However, Dalili et al. (19) did not make such a distinction. Our study design allowed for an evaluation of accuracy while considering possible misattributions, which could explain why our results align more with Bourke et al. (15).

Understanding how childhood maltreatment and depression affect the ability to recognize emotions is important because it can influence how parents interact with their children. Sensitivity is defined as the ability to acknowledge children’s signals, interpret them correctly, and respond appropriately (56). Parental sensitivity significantly affects children’s social and cognitive development. According to a theoretical model elaborated by Leerkes & Augustine (57), which describes the role of emotions in parenting, two main dimensions influence parents’ emotional and physiological arousal, and the regulation necessary to respond appropriately to the child. The first dimension, called the “database”, includes factors like the parent’s developmental history and experience with the child. The second dimension comprises the parent’s emotional well-being, global and trait emotional characteristics, and mood. This model suggests that parental history of maltreatment and depression can both co-occur and interact in a way that affects their ability to recognize and react appropriately to their child’s emotions. Our study offers a validation of that theoretical framework and brings new insights into how a history of maltreatment and depression can interact to create unique profiles in the emotion recognition of children’s facial expressions.

### 4.1 Strengths and limitations

This study sheds light on how depression affects the relationship between childhood maltreatment and parents’ ability to identify facial expressions of emotion in their children. Previous studies examining the interplay of these three variables have used adult faces to assess parents’ emotion recognition skills. To better gauge parents’ true abilities to identify emotions in their child, we used children’s faces as stimuli. Additionally, our study included parents with a wide range of depressive symptoms rather than only those diagnosed with MDD, making our sample more representative of the population. Our research is based on the premise that a history of maltreatment, often accompanied by depressive symptoms, could impair parents’ ability to recognize their children’s emotions, which in turn could affect their parenting behaviors. Further research is needed to investigate how history of maltreatment, depression, and emotion recognition interact to influence parental sensitivity.

Certain limitations in our study are to be acknowledged. Firstly, childhood maltreatment was reported retrospectively, which can be influenced by personal interpretation. Studies have shown that using retrospective and prospective measures of childhood maltreatment can lead to different results, and they cannot be used interchangeably (58). Since retrospective measures are more related to parents’ mental health (Danese & Widom, 2020), and given that our study focuses on parents’ adaptation, the retrospective measure used in our study seems be appropriate. Secondly, that the stimuli used in our study were images of Caucasian children. Research has shown that people can better recognize emotions in individuals who belong to their own ethnic group (59). Therefore, non-Caucasian participants may have had more difficulty recognizing facial expressions. Nonetheless, considering that most of our sample consisted of Caucasian participants and that ethnicity was not correlated to our main variables, this limitation may not significantly affect the study’s results. We also used static faces as stimuli, which allowed us to control perceptual differences between emotional expressions, but it is less realistic than using real-life videos (60). Lastly, the sample size of our study was relatively small, and few fathers were involved. This limits the generalizability of the results. In future studies, it would be relevant to recruit more fathers and reassess whether they exhibit similar patterns of emotion recognition as mothers depending on their maltreatment history and depressive symptoms.

## 5 Conclusion

Parents with a maltreatment history and depression might be more likely to misinterpret their child’s emotional cues, which can affect their ability to respond sensitively when interacting with their child. Therefore, it is crucial to further explore the links between maltreatment, depression, emotion recognition, and parenting behaviors. By providing parents with the necessary tools and resources to recognize and respond to their children’s emotional needs, we could help prevent future instances of maltreatment, thereby positively impacting generations to come.

## Data availability statement

The raw data supporting the conclusions of this article will be made available by the authors, without undue reservation.

## Ethics statement

The studies involving humans were approved by Ethical Committee of the University of Quebec in Outaouais (UQO CER #2020-698). The studies were conducted in accordance with the local legislation and institutional requirements. The participants provided their written informed consent to participate in this study.

## Author contributions

AB: Conceptualization, Data curation, Formal analysis, Funding acquisition, Investigation, Methodology, Project administration, Resources, Supervision, Validation, Visualization, Writing – original draft, Writing – review & editing. RP: Validation, Writing – original draft, Writing – review & editing. CB: Conceptualization, Funding acquisition, Methodology, Validation, Visualization, Writing – original draft, Writing – review & editing.
