# Supplementary material for: Parental depression moderates the relationship between childhood maltreatment and the recognition of children expressions of emotions
Source: Front Psychiatry. 2024 Jun 5;15:1374872. doi: 10.3389/fpsyt.2024.1374872 (PMC11188386; doi:10.3389/fpsyt.2024.1374872)
Supplement: Supplementary file 1 [file Table_1.docx]

**Table S1**

Moderated regression analyses predicting mothers' ability to recognize emotions in children's faces.

|  |  | *b* | SE *b* | 95% CI | | *p* |
| --- | --- | --- | --- | --- | --- | --- |
|  |  |  |  | LL | UL |  |
|  | Constant | .55 | .05 | .45 | .64 | .0000 |
|  | Maltreatment (X1, centered) | .0002 | .004 | -.007 | .01 | .95 |
|  | Depression (X2, centered) | -.0003 | .001 | -.003 | .002 | .81 |
|  | Interaction X1*X2 | .001 | .0002 | .0001 | .001 | .03 |
|  | Education Level | .03 | .01 | .01 | .05 | .005 |

**Table S2**

Moderated regression analyses predicting mothers' ability to recognize sadness in children's faces.

|  |  | *b* | SE *b* | 95% CI | | *p* |
| --- | --- | --- | --- | --- | --- | --- |
|  |  |  |  | LL | UL |  |
|  | Constant | .50 | .07 | .36 | .65 | .0000 |
|  | Maltreatment (X1, centered) | .0001 | .004 | -.01 | .01 | .99 |
|  | Depression (X2, centered) | -.001 | .002 | -.004 | .003 | .71 |
|  | Interaction X1*X2 | .001 | .0004 | .0001 | .002 | .03 |
|  | Education Level | .05 | .02 | .02 | .09 | .003 |
